# Nursing Protocols: Macro and Micropolitics in Primary Health Care Management

**DOI:** 10.1590/0034-7167-2025-0055

**Published:** 2025-12-08

**Authors:** Bruna Coelho, Betina Hörner Schlindwein Meirelles, Fernanda Karla Metelski, Gabriela Marcellino de Melo Lanzoni

**Affiliations:** I Universidade Federal de Santa Catarina. Florianópolis, Santa Catarina, Brazil; II Universidade do Estado de Santa Catarina. Chapecó, Santa Catarina, Brazil

**Keywords:** Population Health Management, Clinical Protocols, Nursing Care, Primary Health Care, Politics., Gestión de la Salud Poblacional, Protocolos Clínicos, Atención de Enfermería, Atención Primaria de Salud, Política.

## Abstract

**Objectives:**

to understand the macro and micropolitical interactions of care management in Primary Health Care regarding the use of nursing protocols.

**Methods:**

qualitative research, anchored in Constructivist Grounded Theory. Thirty healthcare professionals participated in in-depth interviews conducted between 2023 and 2024. Data were analyzed and coded in two stages: initial and focused, resulting in categories and their theoretical relationships.

**Results:**

the main category, “Understanding nursing protocols in macro and micropolitical interactions in Primary Health Care”, emerges from six subcategories that addressed institutional partnerships, strengthening nursing, weaknesses and strengths of Primary Care, evidence-based protocols, review, training, and use of the Practical Approach to Care Kit.

**Final Considerations:**

nursing protocols transcend macro and micropolitical dimensions, expanding clinical practice and promoting care management, with impacts on strengthening primary care and nursing.

## RESUMEN

**Objetivos:**

comprender las interacciones macro y micropolíticas de la gestión de cuidados en Atención Primaria de Salud en relación con el uso de protocolos de enfermería.

**Métodos:**

investigación cualitativa, basada en la Teoría Fundamentada Constructivista. Treinta profesionales de la salud participaron en entrevistas en profundidad realizadas entre 2023 y 2024. Los datos se analizaron y codificaron en dos etapas: inicial y focalizada, lo que resultó en categorías y sus relaciones teóricas.

**Resultados:**

la categoría principal, “Entendiendo los protocolos de enfermería en interacciones macro y micropolíticas en Atención Primaria de Salud”, surge de seis subcategorías que abordaron alianzas institucionales, fortalecimiento de la enfermería, debilidades y fortalezas de la Atención Primaria, protocolos basados en evidencia, revisión, capacitación y uso del Kit de Enfoque Práctico para el Cuidado.

**Consideraciones Finales:**

los protocolos de enfermería trascienden las dimensiones macro y micropolíticas, ampliando la clínica y promoviendo la gestión del cuidado, con impactos en el fortalecimiento de la atención primaria y la enfermería.

## INTRODUCTION

The global healthcare workforce crisis is intensifying. The World Health Organization estimates that by 2035, there will be a global shortage of approximately 12.9 million healthcare professionals, a scenario exacerbated by the COVID-19 pandemic, which has highlighted a shortage of 6 million nursing professionals, exposing the fragility of healthcare systems, especially in developing countries, where professional migration exacerbates the fragility of healthcare systems.

In this scenario, nursing practice is flexible so that expanding the scope of practice and making better use of human resources contribute to more effective care aligned with the population’s needs^(1-3)^. In Brazil, this need aligns with the Primary Health Care (PHC) guidelines, which seeks to overcome the physician-centered model by expanding nurses’ role in problem-solving and user-centered practices. In this context, nursing protocols emerge as essential tools, as they guide care, standardize procedures, reduce care variability, and incorporate technologies, ensuring professional safety and qualified care^(4-6)^.

Despite the Ministry of Health’s efforts to develop clinical protocols and therapeutic guidelines, there is a need for specific nursing documents; this is justified by the country’s regional diversity and the importance of ensuring care safety and quality^(7,8)^. Nursing protocols are tools that organize nurses’ clinical actions, expanding professional autonomy and guiding decision-making. Furthermore, they promote interprofessional integration and contribute to improving the quality of services, strengthening nurses’ role^(4,9)^.

As the main gateway to the health system, PHC is the point in the Health Care Network (In Portuguese, *Rede de Atenção à Saúde* - RAS) where population care management takes place, understood as administrative-assistance actions for organizing the work process, aiming at the quality of direct care to users in healthcare services^(10)^. Therefore, the expansion of nursing clinical practice is fundamental for a more decisive action on the main health conditions present in their territory^(11)^.

The use of multidisciplinary tools also serves as a reference for the organization of primary care services, and an example of this is the Practical Approach to Care Kit (PACK) tool^(12)^. This document was pioneered in the capital of Santa Catarina in 2016, and in 2023, it was expanded from the PACK Florianópolis Program to the PACK Brazil Program. PACK is a tool based on scientific evidence and aligned with national protocols, with a training strategy for professionals, promoting better patient outcomes^(12-14)^.

The use of these clinical protocols in PHC contributes to care management, described by Cecílio^(15)^ as the implementation of health technologies according to people’s needs in different life cycles, aiming to provide well-being, safety, and autonomy for a productive and satisfying life. Care management is understood as a multidimensional, interconnected approach, encompassing the individual, family, professional, organizational, systemic, and societal dimensions.

These dimensions follow macro and micro-spectrum political guidelines. Macro and micropolitics are distinct but not opposing concepts. Thinking about care management from a micropolitical perspective does not exclude the need to also consider it from a macropolitical perspective^(16,17)^. Micropolitics corresponds to the professional and organizational dimensions of care management, characterized by daily activities within the local context. Macropolitics, on the other hand, represents the guidelines, norms, and rules that govern a healthcare facility or institution, such as the Brazilian Health System (In Portuguese, *Sistema Único de Saúde* - SUS) principles and guidelines and regulations for professionals, and is primarily present in the systemic and societal dimensions^(15,16,18)^.

Despite advances in the implementation of nursing protocols in PHC^(5,6,19-24)^, there is a gap in knowledge regarding how these tools interact with the macro and micropolitical dimensions of care management. There is still little discussion about how nursing protocols mediate the relationships between normative guidelines (macropolitics) and daily practices (micropolitics), influencing nurse autonomy and interprofessional integration. Therefore, exploring this topic is justified, since understanding this dynamic can strengthen the organization of healthcare services, optimize care management, and contribute to the effectiveness of PHC, aligning institutional policies with the real needs of the population and professionals.

Based on the above, the question is: how is the relationship between the macro and micropolitics of care management and the use of nursing protocols in PHC?

## OBJECTIVES

To understand the macropolitical and micropolitical interactions of care management in PHC regarding the use of nursing protocols.

## METHODS

### Ethical aspects

The ethical aspects of research involving human subjects were respected, based on Resolutions 466/2012 and 510/2016 of the Brazilian National Health Council, and the research was submitted to and approved by the *Universidade Federal de Santa Catarina* Research Ethics Committee. To ensure participant anonymity, the letter “I” (interviewee) was used, followed by the interview order number (I1, I2, I3, etc.), and G (group), referring to the sample group (G1, G2, G3, etc.). All participants were previously informed about the objectives, risks, and benefits of the study and formally consented by signing the Informed Consent Form.

### Theoretical-methodological framework

The methodological framework adopted was Constructivist Grounded Theory, with a theoretical-philosophical framework of complex thinking proposed by Edgar Morin^(17)^. Data and theories are constructions resulting from interactions and engagement between people, with participants’ perceptions and expressions reflecting reality. The multidimensionality of reality and the importance of interaction between researcher and participant in the process of constructing interpretations are recognized, considering the researcher’s reflexivity in their analyses^(25)^.

### Study design

The research has a qualitative nature, seeking to deepen the understanding of a phenomenon from participants’ perspective^(25)^. The writing of this study followed the Consolidated criteria for REporting Qualitative research checklist guidelines.

### Study setting

The study was conducted in the municipality of Florianópolis, where the municipal health network has 50 Health Centers (HCs) distributed across the four Territorial Support Departments-North, South, Continent, and Center-and provides extensive coverage by Family Health teams. Currently, the municipality has eight institutionalized nursing protocols publicly available on the city’s website. These protocols are managed by the Permanent Commission for Systematization of Nursing Care (CSNC)-specifically, its protocol subcommittee, which is responsible for the ongoing analysis, review, and development of these instruments, aligning them with current health policies, scientific evidence, and local realities. These protocols are implemented in PHC units through periodic training sessions, in line with the territorial organization^(26-28)^.

### Participants and eligibility criteria

The research involved the participation of 30 individuals, divided into three sample groups: 1) 14 nurses working in PHC; 2) seven family and community physicians from PHC; and 3) nine CSNC members and management professionals from the clinic of the Municipal Health Department (MHD) of Florianópolis who are a reference for the PACK team.

Professionals who had at least six months of experience at the time of data collection were included, as it is understood that a period of less than six months is not sufficient for experience and reflection on the use of nursing protocols and care management. Professionals who were not performing their activities regularly due to absences of any nature were excluded.

The HCs for the start of interviews were defined by the Florianópolis MHD, which manages and defines the territories for each study. From there, participants were invited by institutional email or telephone, and all voluntarily accepted the invitation, with no refusals.

### Data collection, organization and analysis

Data collection was conducted by the lead researcher, who was responsible for project design, recruitment, interviews, and data analysis. It is worth noting that the interviewer had completed a qualitative research course and a course on the theoretical framework used, as well as participating in discussions within the research group, which prepared her for conducting interviews. The interviewer had no prior relationship with participants.

The interviews were scheduled according to each person’s availability and preference, taking place both during working hours and at other times, and were conducted in person or via video call (Google Meet^®^ or WhatsApp^®^) between June 2023 and March 2024. In-depth interviews were conducted with a semi-structured script, with the initial question: how do you understand the use of nursing protocols in care management in PHC?

The interviews lasted an average of 25:38 minutes (ranging from 15 to 44 minutes). All interviews were audio-recorded, transcribed in Google Docs^®^, and sent to participants for validation before analysis. NVivo 10^®^ software was used to organize the data, with memo creation and diagrams as an essential intermediate step during analysis.

Data collection and analysis were performed simultaneously, as recommended by the method^(25)^. The analysis was developed through two stages: initial and focused. In the initial stage, each data segment was coded, followed by the focused stage, which examines the most relevant emerging codes with the aim of integrating, summarizing, and structuring the information into axes of analysis or categories and subcategories. From these theoretical relationships between the categories, the emerging theory or phenomenon is revealed^(27,29)^.

Collection ended when theoretical saturation of categories and subcategories was reached, or the collection of new data no longer resulted in new ideas that could contribute to the understanding of the phenomenon^(25)^.

### Data validation

Data validation was performed by sending the transcripts to participants, who made adjustments and contributions, thus ensuring their reliability. Theoretical validation followed Glaser and Strauss’s criteria of adjustment, comprehension, and theoretical generalization^(30)^. Two master’s nurses from the Family Health Strategy, a master’s nurse from the School of Public Health, a PhD nurse from the Florianópolis MHD, and two PhD professionals with expertise in the method and topic studied participated. The process was conducted remotely, with the validation instrument sent via email and/or WhatsApp^®^, according to participants’ preferences. The responses received were analyzed and contributed to improving the final model.

## RESULTS

The three samples totaled 30 professionals, including nurses, physicians, and members of technical committees. The majority (73%) were female, with an average age of 37 and over five years of experience in PHC. Concerning educational background, 60% had a specialization or residency, and 23% had a master’s degree. Data collection was hybrid, with a predominance (83%) of online responses.

**Figure 1:**
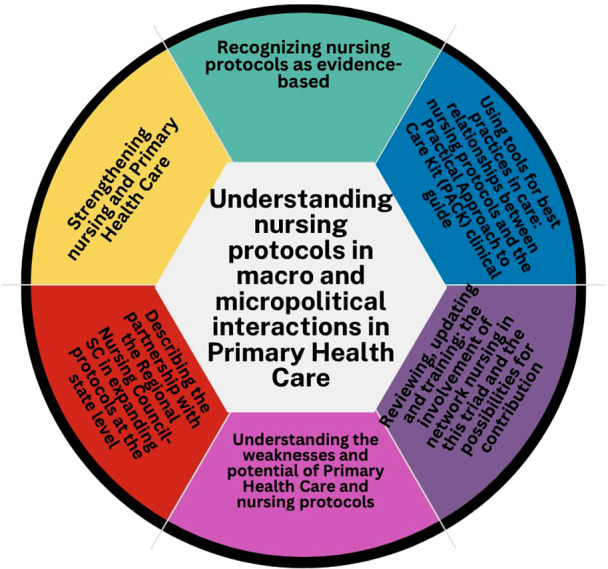
Illustrative representation of the central category and its subcategories

The main category, “Understanding nursing protocols in macro and micropolitical interactions in Primary Health Care”, is supported by six subcategories, represented by Figure 1, like a puzzle in which the pieces interact, connect and integrate to highlight what is happening.

The subcategory “Recognizing nursing protocols as evidence-based” highlights PHC nurses’ recognition of the quality and scientific basis of municipal protocols. They value the fact that they were developed by nurses, focusing on nurse competencies, and appreciate their organization, especially the flowcharts that facilitate guidance for adopting appropriate practices.

*I see the nursing protocol as an extremely empowering tool for nurses’ work in PHC* [...] *it guides us in decision-making, a decision-making process based on more assertive scientific evidence that primarily supports our actions.* (I7G1)

The subcategory “Using tools for best practices in care: relationships between nursing protocols and the Practical Approach to Care Kit (PACK) clinical guide” addresses the development of nursing protocols, which began before PACK’s arrival at MHD. While essential, alignment between protocols and PACK does not always occur simultaneously. The first sample group reported using nursing protocols more than PACK, especially in situations not covered by nursing protocols or to discuss cases with physicians. Despite recognizing the benefits of PACK, one of the interviewees criticized the excessive reliance on protocols.

*Anyone who follows protocol to the letter can’t think outside the box. The moment you can’t see a situation beyond a protocol, you’re becoming stupid* [...]. *What I imagine is that the protocol serves precisely to provide the basis for these clinical conducts. The rest should be inherent to professionals’ conduct* [...]. (I7G1)

The subcategory “Reviewing, updating, and training: the involvement of network nursing in this triad and the possibilities for contribution” highlights the municipal CSNC’s role in reviewing and updating nursing protocols and offering training. Despite the progress, interviewees point to the need for more frequent reviews, aligned with PACK, the new version of which was released in 2023. They acknowledge the committee’s demands and the impact of the pandemic on this process. Regarding training, they suggest greater flexibility, such as the cascade training model used in PACK, with pairs of physicians and nurses training facilitators and local teams, or the provision of video classes on virtual platforms. Some interviewees reported difficulty participating in the courses due to the inability to release from work, which compromises equal access to training opportunities.

*So, we were trained and then we returned from Brasília from this training* [...], *being 45 pairs of physicians and nurses, and these 45 pairs went to their health center to train primary care physicians and nurses in our network* [...] *that’s why we say it’s cascading.* (I7G3)

The CSNC has adopted a strategy of providing a public link on the MHD website, allowing contributions from both internal and external stakeholders. To date, only nurses from the municipal network have participated.

The subcategory “Understanding the weaknesses and potentials in Primary Health Care and nursing protocols” highlights the potential of all the advances achieved by the category, from acquiring a permanent office space for nurses to the autonomy afforded by the expansion of nursing clinical practice. It also highlights professional recognition from both colleagues and patients, and improved access to quality healthcare for the population.

Some criticized the fact that, after implementing the protocols, the university began to work almost exclusively on clinical needs. Another weakness highlighted is the university’s lack of engagement with protocols, which, at times, criticizes but at the same time seems distant from collaboration.

*So, we need to strengthen this* [approach with the university] *and take those who have been doing this* [protocols] *for a long time and start* [...] *the university needs to train people, the university needs to talk to us and bring us into the university to be able to prepare the students, because they come here.* (I3G3)

The subcategory “Describing the partnership with the Regional Nursing Council-SC in expanding protocols at the state level” highlights the partnership with the Regional Nursing Council (In Portuguese, *Conselho Regional de Enfermagem* - Coren) of Santa Catarina to expand Florianópolis’ protocols statewide. It is recognized that Santa Catarina has many small municipalities that would not have the support to develop their own protocols, indicating the need to disseminate the Santa Catarina capital protocols.

*When Coren, in 2016, signed the agreement with us, it provided* [...] *a technical cooperation agreement, where the department* [Florianópolis MHD] *provided the protocols so that Coren/SC could implement this state membership program, via the council.* [...] *we started training people, and it was all in person, in 2018 and 2019. Then the pandemic hit, and we switched to online training.* [...] *I can already tell you that more than 200 municipalities are involved* [...] *the protocol was successful in Florianópolis because of the way it was designed and implemented.* (I4G3)

The final subcategory, “Strengthening nursing and Primary Health Care”, highlights pride in the recognition of nurses’ role in PHC, emphasizing the expansion of their clinical practice, which improves access, promotes autonomy, quaternary prevention, and patient safety. Respondents noted the positive impact of this expansion on the local network and other municipalities, feeling like pioneers in developing nursing protocols before the Federal Nursing Council guidelines, contributing to the document’s development. They also highlighted the expanded scope of practice, such as the insertion of copper intrauterine devices (IUDs), improving access to non-hormonal contraception.

[...] *you can only do training today for IUD insertion, because one day I went to the police station to defend myself, because otherwise the Public Prosecutor’s Office would have banned the practice, and it wasn’t just the practice of IUD insertion, it was the practice of consultation, it was the practice of prescribing medication, of requesting tests. In other words, we opened a path for our profession.* (I4G3)

## DISCUSSION

Nurses play a multifaceted role in the healthcare environment, combining care delivery, administration, research, and teaching, which intertwine and coexist in different contexts in their daily work. The connection between care delivery and nursing management is challenging, but it must be embraced, as it is part of nursing’s exclusive activities, as recommended by the Professional Practice Law^(31)^. Nursing protocols bring advances, but nurses cannot work solely in clinical consultations; they must also perform their other duties. This balance is essential to ensure quality care and the efficiency of healthcare teams.

The term “nursing care management” commonly refers to the integration between the managerial and care areas carried out by nurses^(31,32)^. Cecílio^(15)^ presents the concept of care management in its multiple dimensions, which include: the individual, related to self-care and self-management; the family, which involves actors such as family members, friends, and neighbors, with varying importance depending on the life cycle phase; the professional, which occurs in direct contact between professional and patient, corresponding to the central core of micropolitics; the organizational, which occurs in the healthcare service, with the technical division of labor and where micropolitics is also observed; the systemic, which involves formal and regulated connections, present at the level of health departments, where macropolitics are found; and the societal, represented by the State’s role, governed by macropolitics.

Individual and collective health needs span all dimensions, interconnected in a complex arrangement, with points of interaction that can be controlled by professionals and managers. Therefore, care management must understand these needs in their complexity and diversity^(15,33)^.

As demonstrated by a scoping review^(34)^, the terms “management” and “nursing care management”, although they present clear interfaces, are fundamentally distinguished by their scope of action: care management operates at the macropolitical level, focusing on the RAS’s systemic transformation through strategies such as intersectoral coordination, continuing education, and matrix support, aligned with public policies. Meanwhile, care management operates at the micropolitical level, focusing on the operational organization of healthcare services, with an emphasis on resource planning, team sizing, and optimizing direct patient care. However, as the study highlights, challenges remain in establishing a complete terminological distinction between these concepts^(34)^.

This complexity resonates with Cecílio’s multidimensional approach^(15)^, which, although it does not explicitly use the terms “management” and “administration”, allows for their understanding in different spheres^(15)^. This integrative perspective reveals that, in nursing daily practice, management and administration coexist and complement each other, albeit with different emphases. While the former is oriented toward the system’s structural transformation, i.e., macropolitics, the latter ensures the effectiveness of interventions at the point of user care, i.e., micropolitics.

Macro and micropolitics are interconnected and complementary concepts in care management. In nursing, micropolitics involves challenges such as restricted nurses’ autonomy, resistance to standardization, and lack of adequate training, affecting how these professionals apply protocols in their daily lives. At the macropolitical level, public policies may impose protocols that do not meet the specific needs of certain populations or regions, in addition to being impacted by limited resources and a lack of infrastructure. Excessive bureaucratization can also reduce the efficiency of care, while standardization can generate inequalities in care, depending on patients’ socioeconomic conditions. Protocols must balance uniformity and individualization, focusing on local realities^(6)^.

We can recognize that the SUS and professional council guidelines, standards and regulations constitute the level of health macropolitics^(15,16,18)^. These serve as a basis for developing more specific protocols adapted to local realities. The protocols developed by CSNC are not limited to nurses’ activities, but rather guide the entire nursing team by establishing the limits of their practice. The protocols contribute to a more efficient organization of healthcare work, encompassing not only knowledge for professional practice but also valuing and expanding the recognition of nursing, breaking the physician-centered care paradigm^(22)^.

At the micropolicy level, the daily needs of professionals who provide direct patient care stood out. The need to reassess the protocol training model was highlighted, suggesting a cascade model, with decentralized training or training on virtual platforms. The cascade model allows the knowledge of one trained team to be propagated to others, reaching different hierarchical levels and resulting in a broader impact^(35)^. According to Hoffmann^(36)^, the use of virtual platforms facilitates access for participants from different regions, something that is difficult in in-person courses. Furthermore, the material available online allows for study at each participant’s own pace.

It is crucial that municipal managers recognize the importance of protocols and ensure their continuous updating. According to Cornick^(37)^, PACK should be updated annually, but reports indicate that there was a gap between 2020 and 2023 without revisions. These tools have significant impacts in helping translate scientific knowledge into professional care practice, supporting decision-making^(38)^.

The capital’s nursing protocols had such a positive impact that the clinical practice identified at the micropolicy level resulted in a partnership with Coren/SC, enabling the dissemination of the material throughout the state of Santa Catarina. The goal is to comply with the Nursing Professional Practice Law, protecting nursing clinical practice, optimizing work, promoting comprehensive care, training for analysis and decision-making, aligning practice with national and international guidelines, and improving PHC^(6,39)^.

The implementation of protocols at the state and national levels is considered positive by participants, as it allows managers from different regions of the country to identify technological and human needs, promoting advances at a macro level. One example cited is the PACK protocol, which recommends, in cases of seizures, performing a glucose test with a glucometer. This requires municipalities without the equipment to provide it, aligning practices. The variations in the quality of care across the country represent a challenge that can be addressed by adapting and using clinical support tools for professionals^(38)^.

The use of PACK promotes the expansion of the scope of PHC services, the training of healthcare professionals, and the provision of practical tools to keep them up to date with evidence and ministerial policies. This initiative was part of a comprehensive primary care reform, which includes improved access, local nurse prescribing protocols, and standardization of clinical delivery^(14)^.

These changes, generated by demands in the systemic dimension, are reflected in both the professional and organizational dimensions, as well as the societal dimension, impacting the entire health system. They highlight this macro and micropolitical interaction, which influences and transforms one another simultaneously. The result is stronger professional categories and a more effective PHC system, adapted to the context and capable of responding to the population’s health demands with quality, punctuality, individuality, uniqueness, and complexity^(17,33)^.

The expansion of clinical nursing practice in PHC, in an interprofessional environment, results from factors such as the broadening of the scope of practice, driven by changes in population profiles, and the evolution of care and work models. This practice, guided by health needs and agility in care, adopts a comprehensive approach, centered on the person, family, and community, going beyond the strictly biomedical model^(39)^.

In today’s PHC system, care cannot be restricted to a single professional category. Sharing responsibilities is essential to meet the growing population’s health demands, exacerbated by the scarcity of human resources. This reality requires expanding nursing clinical practice and adopting advanced practices to adequately respond to needs^(1-3)^.

In 2017, with the new Brazilian National Primary Care Policy, nursing faced a nationwide legal battle over the request for tests and medication prescriptions by nurses in PHC, in a lawsuit filed by the Federal Council of Medicine. This case highlighted that one of the main obstacles to interprofessional practice is corporate disputes, which prioritize segmented interests over the common good of professionals, managers, patients, and the population^(40)^.

Nursing, the SUS, and the audience face challenges with the acceptance of prescriptions issued by nurses in private pharmacies, including those under the *Farmácia Popular* (Popular Pharmacy) program. Although authorized to prescribe medications based on ministerial and municipal protocols, nursing prescriptions are only accepted in pharmacies affiliated with public services through a specific administrative act, and are not accepted by private pharmacies^(41)^. To resolve this impasse, the Federal District approved Bill 574/2023, which requires private pharmacies to accept prescriptions from nurses in public health programs and routinely authorized by healthcare institutions. At the national level, Bill 3,949/23, currently under consideration, aims to extend this requirement nationwide and regulate test requests made by nurses^(42)^. These are battles and achievements that not only strengthen nursing, but also the SUS, directly benefiting public health and the population.

### Study limitations

During the research, difficulties were encountered in recruiting participants, which required expanding data collection sites and collaborating with coordinators and others involved in services for dissemination. Another limitation of the research was the lack of data collection from healthcare system users, which prevented us from understanding their perceptions of the expansion of nursing practice in PHC and the impact of nursing protocols on their care. Therefore, it is recommended that future studies include this population group to gain a broader understanding of the topic.

### Contributions to nursing

This study is believed to contribute to nursing by strengthening care management models and promoting evidence-based practices. The results highlight the complex interaction between nursing protocols and macro and micropolitics, highlighting the need for continuous updating of protocols in line with other health programs and public policies.

## FINAL CONSIDERATIONS

The relationship between macro and micropolitics for care management in PHC occurs in a complementary manner, through nursing protocols, in a dynamic and interdependent process amid interactions between professionals and the multiple aspects of healthcare. Recognizing the relationship between macro and micropolitics in care management is essential for nursing to expand its autonomy, representation, and recognition as a profession.

Care management in PHC encompasses all dimensions, from individual to societal, with the presence of nursing protocols. PACK uses nursing protocols for its own improvement, and both interact in a context where everything is interconnected.

The expansion of nursing clinical practice has a direct impact on care management, and is achieved through the use of nursing protocols in PHC, understanding that their use is not done in isolation, but is done in nurses’ consultation, through the nursing process, as well as subsidizing the clinical discussion with other professionals.

## Data Availability

The research data are available only upon request.
