# Supplementary material for: Nursing Protocols: Macro and Micropolitics in Primary Health Care Management
Source: Rev Bras Enferm. 2025 Dec 8;78(6):e20250055. doi: 10.1590/0034-7167-2025-0055 (PMC12707609; doi:10.1590/0034-7167-2025-0055)
Supplement: Translation

# Translation: Protocolos de Enfermagem: Macro e Micropolíticas na Gestão do Cuidado da Atenção Primária à Saúde

**DOI:** 10.1590/0034-7167-2025-0055pt

**Authors:** Bruna Coelho, Betina Hörner Schlindwein Meirelles, Fernanda Karla Metelski, Gabriela Marcellino de Melo Lanzoni

**Affiliations:** I Universidade Federal de Santa Catarina. Florianópolis, Santa Catarina, Brasil; II Universidade do Estado de Santa Catarina. Chapecó, Santa Catarina, Brasil

**Keywords:** Gestão da Saúde da População, Protocolos Clínicos, Cuidados de Enfermagem, Atenção Primária à, Saúde, Política.

## Abstract

**Objetivos:**

compreender as interações macro e micropolíticas da gestão do cuidado na Atenção Primária à Saúde quanto ao uso dos protocolos de enfermagem.

**Métodos:**

pesquisa qualitativa, ancorada na Teoria Fundamentada nos Dados Construtivista. Participaram 30 profissionais de saúde por meio de entrevistas em profundidade, realizadas entre 2023 e 2024. Os dados foram analisados e codificados em duas etapas, inicial e focalizada, originando as categorias e suas relações teóricas.

**Resultados:**

a categoria principal, “Percebendo os protocolos de enfermagem nas interações macro e micropolíticas na Atenção Primária à Saúde”, emerge de seis subcategorias que abordaram parcerias institucionais, fortalecimento da enfermagem, fragilidades e potencialidades da Atenção Primária, protocolos baseados em evidências, revisão, capacitação, e utilização do *Practical Approach to Care Kit*.

**Considerações Finais:**

os protocolos de enfermagem transpassam dimensões macro e micropolíticas, ampliando a clínica e promovendo a gestão e gerenciamento do cuidado, com impactos no fortalecimento da atenção primária e da enfermagem.

## INTRODUÇÃO

A crise global de profissionais de saúde está se intensificando. A Organização Mundial da Saúde estima que, até 2035, haverá um déficit mundial de cerca de 12,9 milhões de profissionais de saúde, cenário agravado pela pandemia de COVID-19, que evidenciou a escassez de 6 milhões de profissionais de enfermagem, expondo a fragilidade dos sistemas de saúde, sobretudo em países em desenvolvimento, onde a migração profissional agrava a fragilidade dos sistemas de saúde.

Diante desse cenário, a atuação da enfermagem é flexível, de modo que a ampliação do escopo de prática e o melhor aproveitamento dos recursos humanos contribuem para uma assistência mais eficaz e alinhada às necessidades da população^(1-3)^. No Brasil, essa necessidade se alinha às diretrizes da Atenção Primária à Saúde (APS), que busca superar o modelo médico-centrado ao ampliar o papel do enfermeiro em práticas resolutivas e centradas no usuário. Nesse contexto, os protocolos de enfermagem emergem como ferramentas essenciais, pois norteiam o cuidado, padronizam procedimentos, reduzem variabilidades assistenciais e incorporam tecnologias, garantindo segurança ao profissional e qualificação ao atendimento^(4-6)^.

Apesar dos esforços do Ministério da Saúde na elaboração de protocolos clínicos e diretrizes terapêuticas, há necessidade de documentos específicos para a enfermagem; isso se justifica pela diversidade regional do país e pela importância de garantir a segurança e qualidade do cuidado^(7,8)^. Os protocolos de enfermagem são ferramentas que organizam as ações clínicas dos enfermeiros, ampliando a autonomia profissional e orientando a tomada de decisão. Além disso, favorecem a integração interprofissional e contribuem para a melhoria da qualidade dos serviços, fortalecendo a atuação do enfermeiro^(4,9)^.

Como principal porta de entrada ao sistema de saúde, a APS é o ponto na Rede de Atenção à Saúde (RAS), onde ocorre o gerenciamento do cuidado da população, compreendido como as ações administrativo-assistenciais para organização do processo de trabalho, visando à qualidade do cuidado direto ao usuário nos serviços de saúde^(10)^. Desta forma, a expansão da prática clínica do enfermeiro é fundamental para uma atuação mais resolutiva sobre as principais condições de saúde presentes em seu território^(11)^.

O uso de ferramentas multiprofissionais também serve como referência para a organização dos serviços de atenção primária, e um exemplo disso é a ferramenta *Practical Approach to Care Kit* (PACK)/*Kit* de Cuidados em Atenção Primária^(12)^. Este documento foi implementado de forma pioneira na capital catarinense no ano de 2016, e em 2023, teve sua expansão, passando de Programa PACK Florianópolis para Programa PACK Brasil. O PACK é uma ferramenta baseada em evidências científicas e alinhada aos protocolos nacionais, com uma estratégia de treinamento para profissionais, promovendo melhores resultados para os pacientes^(12-14)^.

O uso desses protocolos clínicos na APS contribui para a gestão do cuidado, descrita por Cecílio^(15)^ como a viabilização de tecnologias de saúde de acordo com as necessidades das pessoas nos diferentes ciclos de vida, visando proporcionar bem-estar, segurança e autonomia para uma vida produtiva e satisfatória. A gestão do cuidado é compreendida de forma multidimensional, conectada entre si, abrangendo as dimensões individual, familiar, profissional, organizacional, sistêmica e societária.

Essas dimensões seguem diretrizes políticas de macro e micro espectro. Macro e micropolítica são conceitos distintos, mas não opostos. Pensar em gestão do cuidado do ponto de vista da micropolítica não exclui a necessidade de pensá-la igualmente de um ponto de vista da macropolítica^(16,17)^. A micropolítica corresponde às dimensões profissional e organizacional de gestão do cuidado, sendo caracterizada pela atividade cotidiana a partir do contexto local. Já a macropolítica representa as diretrizes, normas e regras que determinam um estabelecimento e instituição de saúde, como os princípios e diretrizes do Sistema Único de Saúde (SUS) e regulações dos profissionais, estando presentes principalmente nas dimensões sistêmica e societária^(15,16,18)^.

Apesar dos avanços na implementação de protocolos de enfermagem na APS^(5,6,19-24)^, observa-se uma lacuna no conhecimento sobre como essas ferramentas se articulam com as dimensões macro e micropolítica da gestão do cuidado. Ainda há pouca discussão sobre como os protocolos de enfermagem medeiam as relações entre as diretrizes normativas (macropolíticas) e as práticas cotidianas (micropolíticas), influenciando a autonomia do enfermeiro e a integração interprofissional. Justifica-se, portanto, a exploração dessa temática, uma vez que compreender essa dinâmica pode fortalecer a organização dos serviços de saúde, otimizar a gestão do cuidado e contribuir para a resolutividade da APS, alinhando as políticas institucionais às necessidades reais da população e dos profissionais.

Com base no exposto, questiona-se: como se dá a relação entre as macro e micropolíticas da gestão do cuidado e o uso dos protocolos de enfermagem na APS?

## OBJETIVOS

Compreender as interações macropolíticas e micropolíticas da gestão do cuidado na APS quanto ao uso dos protocolos de enfermagem.

## MÉTODOS

### Aspectos éticos

Os aspectos éticos da pesquisa que envolve seres humanos foram respeitados, fundamentados nas Resoluções n^o^ 466/2012 e n^o^ 510/2016 do Conselho Nacional de Saúde, sendo a pesquisa submetida e aprovada pelo Comitê de Ética em Pesquisa com Seres Humanos da Universidade Federal de Santa Catarina. Para garantir anonimato dos participantes, foram utilizadas a letra “E” (entrevistado), seguida do número de ordem de realização da entrevista (E1, E2, E3…), e G (grupo), referente ao grupo amostral (G1, G2, G3…). Todos os participantes foram previamente informados sobre os objetivos, riscos e benefícios do estudo, consentindo formalmente por meio da assinatura do Termo de Consentimento Livre e Esclarecido.

### Referencial teórico-metodológico

O referencial metodológico adotado foi a Teoria Fundamentada nos Dados Construtivista, com referencial teórico-filosófico do pensamento complexo proposto por Edgar Morin^(17)^. Os dados e as teorias são construções resultantes das interações e do envolvimento entre as pessoas, com as percepções e expressões dos participantes refletindo a realidade. Reconhecem-se a multidimensionalidade da realidade e a importância da interação entre pesquisador e participante no processo de construção das interpretações, considerando a reflexividade do pesquisador em suas análises^(25)^.

### Tipo de estudo

A pesquisa possui um caráter qualitativo, buscando aprofundar a compreensão de um fenômeno a partir da perspectiva dos participantes^(25)^. A redação deste estudo seguiu as diretrizes do *checklist COnsolidated criteria for REporting Qualitative research* para estudos qualitativos.

### Cenário do estudo

O estudo foi realizado no município de Florianópolis, onde a rede municipal de saúde conta com 50 Centros de Saúde (CS), distribuídos pelos quatro Departamentos de Apoio Territorial - Norte, Sul, Continente e Centro -, apresentando ampla cobertura de equipes de Saúde da Família. Atualmente, o município possui oito protocolos de enfermagem institucionalizados e disponíveis publicamente no portal da prefeitura, gerenciados pela Comissão Permanente para a Sistematização da Enfermagem (CSAE) - especificamente por sua subcomissão de protocolos, responsável pela análise, revisão e elaboração contínua desses instrumentos, alinhando-os às políticas de saúde vigentes, evidências científicas e realidades locais. Esses protocolos são implementados nas unidades de APS através de capacitações periódicas, em consonância com a organização territorial^(26-28)^.

### Participantes e critérios de elegibilidade

A pesquisa envolveu a participação de 30 indivíduos, divididos em três grupos amostrais: 1) 14 enfermeiros que atuam na APS; 2) sete médicos de família e comunidade da APS; e 3) nove membros da CSAE e profissionais da gestão da clínica da Secretaria Municipal de Saúde (SMS) de Florianópolis que são referência da equipe do PACK.

Considerou-se como critério de inclusão para participação do estudo os profissionais que possuíam, minimamente, seis meses de experiência em relação à data de coleta de dados, pois entende-se que um período inferior a seis meses não é suficiente para a vivência e reflexão acerca do uso dos protocolos de enfermagem e gestão do cuidado. Considerou-se como como critério de exclusão os profissionais que não estavam desenvolvendo suas atividades de forma regular em decorrência de afastamentos de qualquer natureza.

Os CS para o início das entrevistas foram definidos pela SMS de Florianópolis, que gerencia e define os territórios de cada pesquisa. A partir disso, os participantes foram convidados por e-mail institucional ou telefone, e todos aceitaram voluntariamente o convite, não havendo recusas.

### Coleta, organização e análise dos dados

A coleta de dados foi conduzida pela pesquisadora principal, responsável pela elaboração do projeto, recrutamento, entrevistas e análise dos dados. Destaca-se que a entrevistadora cursou disciplina de pesquisa qualitativa e disciplina sobre o referencial teórico utilizado, além de participação em discussões no grupo de pesquisa, o que a preparou para a condução de entrevistas. A entrevistadora não tinha relação prévia com os participantes.

As entrevistas foram agendadas conforme disponibilidade e preferência de cada um, ocorrendo tanto durante o horário de trabalho quanto em outros momentos, e realizadas presencialmente ou por videochamada (*Google Meet^®^* ou *WhatsApp^®^*) entre junho de 2023 e março de 2024. Foram realizadas entrevistas em profundidade com roteiro semiestruturado, tendo como pergunta inicial: como você significa o uso dos protocolos de enfermagem na gestão do cuidado na APS?

As entrevistas tiveram duração média de 25:38 minutos (variando entre 15 e 44 minutos). Todas foram audiogravadas, transcritas no *Google Docs^®^* e enviadas aos participantes para validação antes da análise. Para a organização dos dados, utilizou-se o *software NVivo 10^®^*, com elaboração de memorandos e diagramas como etapa intermediária essencial durante a análise.

A coleta e análise dos dados foi realizada simultaneamente, conforme recomendação do método^(25)^. A análise foi desenvolvida por meio de duas etapas: inicial e focalizada. Na etapa inicial, foi realizada a codificação de cada segmento de dado, seguida da etapa focalizada, que examina os códigos emergentes mais relevantes com o objetivo de integrar, resumir e estruturar as informações em eixos de análise ou categorias e subcategorias. A partir dessas relações teóricas entre as categorias, revela-se a teoria ou fenômeno emergente^(27,29)^.

A coleta finalizou quando se atingiu a saturação teórica das categorias e subcategorias, ou a coleta de novos dados não resultava mais em novas ideias que pudessem contribuir para a compreensão do fenômeno^(25)^.

### Validação dos dados

A validação dos dados foi realizada com o envio das transcrições aos participantes, que realizaram ajustes e contribuições, garantindo, assim, a sua fidedignidade. A validação teórica seguiu os critérios de ajuste, compreensão e generalização teórica de Glaser e Strauss^(30)^. Participaram duas enfermeiras mestres da Estratégia Saúde da Família, uma enfermeira mestre da Escola de Saúde Pública, uma enfermeira doutora da SMS de Florianópolis, e duas doutoras com *expertise* no método e na temática estudada. O processo foi realizado remotamente, com envio do instrumento de validação por e-mail e/ou *WhatsApp* ^®^, conforme a preferência dos participantes. As respostas recebidas foram analisadas e contribuíram para aperfeiçoar o modelo final.

## RESULTADOS

As três amostras totalizaram 30 profissionais, incluindo enfermeiros, médicos e membros de comissões técnicas. A maioria (73%) era do sexo feminino, com idade média de 37 anos e experiência na APS superior a cinco anos. Quanto à formação, 60% possuíam especialização *lato sensu* ou residência, e 23% possuíam mestrado. A coleta de dados ocorreu de forma híbrida, com predominância (83%) de respostas *online*.

A categoria principal, “Percebendo os protocolos de enfermagem nas interações macro e micropolíticas na Atenção Primária à Saúde”, é sustentada por seis subcategorias, representadas pela Figura 1, como um quebra-cabeça em que as peças interagem, conectam e integram para evidenciar o que está acontecendo.

**Figura 1:** Representação ilustrativa da categoria central e suas subcategorias

A subcategoria “Reconhecendo os protocolos de enfermagem como baseados em evidência” destaca o reconhecimento dos enfermeiros da APS quanto à qualidade e fundamentação científica dos protocolos municipais. Eles valorizam o fato de serem desenvolvidos por enfermeiros, com foco nas competências da categoria, e apreciam sua organização, especialmente os fluxogramas que facilitam o direcionamento para adoção de condutas adequadas.

*Eu vejo o protocolo de enfermagem como uma ferramenta extremamente potencializadora do trabalho do enfermeiro na APS* [...] *nos direciona na tomada de decisão, uma tomada de decisão baseada em evidências científicas mais assertivas e que respalda principalmente a nossa atuação.* (E7G1)

A subcategoria “Utilizando ferramentas para melhores práticas no cuidado: relações entre protocolos de enfermagem e guia clínico *Practical Approach to Care Kit (PACK)*” aborda o desenvolvimento dos protocolos de enfermagem, iniciado antes da chegada do PACK à SMS. Apesar de essencial, o alinhamento entre os protocolos e o PACK nem sempre ocorre simultaneamente. O primeiro grupo amostral relatou utilizar mais os protocolos de enfermagem do que o PACK, principalmente em situações não contempladas pelos protocolos de enfermagem ou para discutir casos com médicos. Apesar de reconhecer os benefícios do PACK, uma das entrevistadas criticou a dependência excessiva dos protocolos.

*Qualquer um que segue o protocolo à risca não consegue pensar fora daquilo. A partir do momento que você não consegue olhar uma situação além de um protocolo, você está ficando burro* [...]. *O que eu imagino é que o protocolo venha exatamente para dar base para essas condutas clínicas. O restante deveria ser inerente à conduta do profissional* [...]. (E7G1)

A subcategoria “Revisando, atualizando e capacitando: o envolvimento da enfermagem da rede nessa tríade e as possibilidades para contribuição” destaca o papel da CSAE municipal na revisão, atualização dos protocolos de enfermagem e oferta de capacitações. Apesar dos avanços, os entrevistados apontam a necessidade de revisões mais frequentes, alinhadas ao PACK, cuja nova versão foi lançada em 2023. Reconhecem as demandas da comissão e o impacto da pandemia nesse processo. Sobre as capacitações, sugerem maior flexibilidade, como o modelo de treinamento em cascata usado no PACK, com duplas de médicos e enfermeiros treinando facilitadores e equipes locais, ou a disponibilização de videoaulas em plataformas virtuais. Alguns entrevistados relataram dificuldade em participar dos cursos devido à impossibilidade de liberação do trabalho, o que compromete o acesso igualitário aos momentos de formação.

*Então, a gente foi treinado e aí a gente voltou lá de Brasília deste* treinamento [...], *sendo 45 duplas, de médicos e enfermeiros, e essas 45 duplas foram para o seu centro de saúde para treinar os médicos e enfermeiros da atenção primária da nossa rede* [...] *por isso que a gente diz que é em cascata.* (E7G3)

A CSAE adotou como estratégia disponibilizar um *link* público no site da SMS, permitindo contribuições de pessoas internas e externas à rede. Até o momento, participaram apenas enfermeiros da rede municipal.

A subcategoria “Percebendo as fragilidades e potencialidade na Atenção Primária à Saúde e dos protocolos de enfermagem” aponta como potencialidade todos os avanços conquistados pela categoria, desde conquista de uma sala para consultório fixo para o enfermeiro até a autonomia proporcionada pela ampliação da clínica do enfermeiro. Também destacam o reconhecimento profissional, tanto por colegas de equipe quanto pelos pacientes, e melhoria do acesso a serviço de saúde de qualidade à população.

Houve quem criticou que, após a implementação dos protocolos, passou a atuar quase exclusivamente atendendo demandas clínicas. Outra fragilidade destacada é a falta de aproximação da universidade com os protocolos que, em alguns momentos, fazem críticas, mas ao mesmo tempo se mostram distantes a colaborar.

*Então, a gente precisa fortalecer isso* [aproximação com a universidade] *e pegar quem está fazendo isso* [protocolos] *há muito tempo e começar* [...] *a universidade precisa capacitar as pessoas, a universidade precisa conversar com a gente e levar a gente para dentro da universidade para poder preparar os alunos, porque eles vêm para cá.* (E3G3)

A subcategoria “Descrevendo a parceria com Conselho Regional de Enfermagem-SC na ampliação dos protocolos a nível estadual” aponta a parceria realizada com o Conselho Regional de Enfermagem (Coren)/SC para expandir os protocolos de Florianópolis para todo o estado. Reconhece-se que Santa Catarina tem muitos municípios pequenos, os quais não teriam suporte para elaboração de protocolo próprio, indicando a necessidade de difundir os protocolos da capital catarinense.

*Quando o Coren, em 2016, assina o termo com a gente, propicia* [...] *um termo de cooperação técnica, onde a secretaria* [SMS de Florianópolis] *cede os protocolos para que o Coren/SC faça esse programa de adesão do Estado, via conselho*. [...] *começamos a treinar a galera e era tudo presencial, em 2018 e 2019. Aí veio a pandemia, a gente mudou para o treinamento online*. [...] *já posso te dizer que são mais de 200 municípios envolvidos* [...] *o protocolo deu certo em Florianópolis pela forma como ele foi construído e pela forma como ele foi implementado.* (E4G3)

A última subcategoria, “Fortalecendo a enfermagem e a Atenção Primária à Saúde”, destaca o orgulho pelo reconhecimento do papel do enfermeiro na APS, enfatizando a ampliação de sua prática clínica, que melhora o acesso, promove autonomia, prevenção quaternária e segurança do paciente. Os entrevistados percebem o impacto positivo dessa expansão na rede local e em outros municípios, sentindo-se pioneiros no desenvolvimento de protocolos de enfermagem antes das diretrizes do Conselho Federal de Enfermagem, com contribuição na elaboração do documento. Também destacam a ampliação do escopo de prática, como a inserção de dispositivo intrauterino (DIU) de cobre, melhorando o acesso ao método contraceptivo não hormonal.

[...] *só pode fazer treinamento hoje para inserção de DIU, porque um dia eu fui para a delegacia defender, porque senão o Ministério Público tinha proibido a prática, e não era só a prática da inserção de DIU, era prática da consulta, era prática de prescrição do medicamento, a de solicitação de exame. Quer dizer, a gente abriu um caminho para nossa profissão.* (E4G3)

## DISCUSSÃO

O enfermeiro desempenha um papel multifacetado no ambiente de saúde, combinando a prestação de cuidados, administração, pesquisa e ensino, que se entrelaçam e coexistem em diferentes contextos no dia a dia de trabalho. A conexão entre a prestação de cuidados e a gerência do enfermeiro constitui um cenário desafiador, mas deve ser assumido, pois faz parte das atividades privativas da categoria, conforme preconizado na Lei do Exercício Profissional^(31)^. Os protocolos de enfermagem trazem avanços, mas o enfermeiro não pode atuar apenas na consulta clínica, pois ele também deve realizar suas demais atribuições. Esse equilíbrio é essencial para garantir a qualidade do atendimento e a eficiência das equipes de saúde.

O termo “gestão do cuidado na enfermagem” comumente se refere à integração entre as áreas gerencial e assistencial realizadas pelo enfermeiro^(31,32)^. Cecílio^(15)^ apresenta o conceito de gestão do cuidado em suas múltiplas dimensões, que incluem: a individual, relacionada ao cuidar de si e à autogestão; a familiar, que envolve atores como familiares, amigos e vizinhos, com importância variável conforme a fase do ciclo de vida; a profissional, que ocorre no contato direto entre profissional e paciente, correspondendo ao núcleo central da micropolítica; a organizacional, que se dá no serviço de saúde, com a divisão técnica do trabalho e onde também se observa a micropolítica; a sistêmica, que envolve conexões formais e regulamentadas, presentes a nível de secretarias de saúde, onde se encontram as macropolíticas; e a societária, representada pelo papel do Estado, regido pelas macropolíticas.

As necessidades de saúde, individuais e coletivas, passam por todas as dimensões, interconectadas na forma de um arranjo complexo, com pontos de interação que podem ser controlados por profissionais e gestores. Assim, a gestão do cuidado deve compreender essas necessidades em sua complexidade e diversidade^(15,33)^.

Como demonstra revisão de escopo^(34)^, os termos “gestão” e “gerenciamento do cuidado de enfermagem”, embora apresentem interfaces evidentes, distinguem-se fundamentalmente por seu escopo de atuação: a gestão do cuidado opera no nível macropolítico, com foco na transformação sistêmica da RAS, mediante estratégias como articulação intersetorial, educação permanente e matriciamento, alinhadas às políticas públicas. Enquanto isso, o gerenciamento do cuidado atua no nível micropolítico, voltando-se à organização operacional do serviço de saúde, com ênfase no planejamento de recursos, dimensionamento de equipes e otimização do cuidado direto ao usuário. Contudo, como ressalta o estudo, ainda persistem desafios para uma distinção terminológica absoluta entre esses conceitos^(34)^.

Essa complexidade encontra ressonância na abordagem multidimensional de Cecílio^(15)^ que, embora não empregue explicitamente os termos “gestão” e “gerenciamento”, permite a compreensão destes em diferentes esferas^(15)^. Nessa perspectiva integradora, revela que, na prática cotidiana do enfermeiro, gestão e gerenciamento coexistem e se complementam, ainda que com ênfases distintas. Enquanto a primeira se orienta para a transformação estrutural do sistema, ou seja, macropolítica, a segunda assegura a efetividade das intervenções no ponto de atenção ao usuário, ou seja, micropolítica.

Macro e micropolítica são conceitos interligados e complementares na gestão do cuidado. Na enfermagem, a micropolítica envolve desafios como restrição à autonomia dos enfermeiros, resistência à padronização e falta de capacitação adequada, afetando a forma como esses profissionais aplicam os protocolos no cotidiano. No nível macropolítico, políticas públicas podem impor protocolos que não atendem às necessidades específicas de certas populações ou regiões, além de serem impactadas por recursos limitados e falta de infraestrutura. A burocratização excessiva também pode reduzir a eficiência do cuidado, enquanto a padronização pode gerar desigualdades no atendimento, dependendo das condições socioeconômicas dos pacientes. Protocolos devem equilibrar uniformidade e individualização, com foco na realidade local^(6)^.

Podemos reconhecer que as diretrizes, normas e regulamentações do SUS e dos conselhos profissionais constituem o nível das macropolíticas de saúde^(15,16,18)^. Esses servem como base para a elaboração de protocolos mais específicos, adaptados às realidades locais. Os protocolos desenvolvidos pela CSAE não se limitam às atividades do enfermeiro, mas sim orientam toda a equipe de enfermagem ao estabelecer os limites de atuação, os protocolos contribuem para uma organização mais eficiente do trabalho em saúde, contemplando não apenas conhecimento para a prática profissional, mas também valorizando e ampliando o reconhecimento da categoria, rompendo o paradigma do cuidado centrado no médico^(22)^.

No nível da micropolítica, destacam-se as necessidades diárias dos profissionais que prestam assistência direta ao paciente. Foi evidenciada a necessidade de reavaliar o modelo de capacitação dos protocolos, sugerindo um modelo em cascata, com treinamento descentralizado ou em plataformas virtuais. O modelo em cascata permite que o conhecimento de uma equipe treinada seja propagado para outras, alcançando níveis hierárquicos diversos e resultando em impacto mais amplo^(35)^. Já o uso de plataformas virtuais, segundo Hoffmann^(36)^, facilita o acesso de participantes de diferentes regiões, algo difícil em cursos presenciais. Além disso, o material disponível *online* permite estudos no tempo de cada participante.

É fundamental que gestores municipais reconheçam a importância dos protocolos e garantam sua atualização contínua. Segundo Cornick^(37)^, o PACK deveria ser atualizado anualmente, mas, conforme os relatos, houve um intervalo de 2020 a 2023 sem revisões. Estas ferramentas causam impactos importantes no auxílio de tradução do conhecimento científico para a prática profissional assistencial, sendo suporte para tomada de decisão^(38)^.

Os protocolos de enfermagem da capital tiveram impacto tão positivo que a prática clínica identificada a nível das micropolíticas resultou em parceria com o Coren/SC, permitindo a disseminação do material em todo o estado de Santa Catarina. Seu objetivo é cumprir com a Lei do Exercício Profissional de Enfermagem, protegendo a prática clínica do enfermeiro, otimizar o trabalho, promover atendimento integral, capacitar para análise e decisão, alinhar a prática às diretrizes nacionais e internacionais, e aprimorar a APS^(6,39)^.

A implementação de protocolos em nível estadual e nacional é considerada positiva pelos participantes, pois permite que gestores de diferentes regiões do país identifiquem necessidades tecnológicas e humanas, promovendo avanços em âmbito macro. Um exemplo citado é o PACK, que recomenda, em casos de convulsão, a realização de teste de glicose com glicosímetro. Isso obriga municípios sem o equipamento a providenciá-lo, alinhando práticas. As variações na qualidade dos cuidados no país representam um desafio que pode ser enfrentado pela adaptação e uso de ferramentas de suporte clínico para os profissionais^(38)^.

O uso do PACK promove a ampliação do escopo dos serviços de APS, a qualificação dos profissionais de saúde e o fornecimento de ferramentas práticas para mantê-los atualizados com as evidências e políticas ministeriais. Essa iniciativa fez parte de uma reforma abrangente da atenção primária, que inclui acesso avançado, protocolos locais para prescrição de enfermeiros e padronização da oferta clínica^(14)^.

Essas mudanças, geradas pelas demandas na dimensão sistêmica, refletem tanto nas dimensões profissional e organizacional quanto na dimensão societária, impactando toda a organização do sistema de saúde. Elas tornam visível essa interação macro e micropolítica, que se influenciam e se transformam simultaneamente. Como resultado, surgem categorias profissionais mais fortalecidas e uma APS mais resolutiva, adaptada ao contexto e capaz de responder às demandas de saúde da população com qualidade, pontualidade, individualidade, singularidade e complexidade^(17,33)^.

A expansão da prática clínica da enfermagem na APS, em um ambiente interprofissional, resulta de fatores como a ampliação do escopo de atuação, impulsionada pelas mudanças nos perfis populacionais, e a evolução dos modelos de atenção e trabalho. Essa prática, orientada pelas necessidades de saúde e pela agilidade no atendimento, adota uma abordagem integral, centrada na pessoa, família e comunidade, indo além do modelo estritamente biomédico^(39)^.

Na APS atual, o cuidado não pode ser restrito a uma única categoria profissional. O compartilhamento de responsabilidades é essencial para atender às crescentes demandas de saúde da população, agravadas pela escassez de recursos humanos. Essa realidade exige a ampliação da prática clínica do enfermeiro e a adoção de práticas avançadas para responder adequadamente às necessidades^(1-3)^.

Em 2017, com a nova Política Nacional de Atenção Básica, a enfermagem enfrentou um embate jurídico nacional sobre a solicitação de exames e prescrição de medicamentos por enfermeiros na APS em ação movida pelo Conselho Federal de Medicina. Esse caso evidenciou que um dos principais obstáculos à prática interprofissional são as disputas corporativas, que priorizam interesses segmentados em detrimento do bem comum de profissionais, gestores, usuários e da população^(40)^.

A enfermagem, o SUS e a população enfrentam desafios com a aceitação de receitas emitidas por enfermeiros em farmácias privadas, incluindo as do programa Farmácia Popular. Apesar de autorizados a prescrever medicamentos com base em protocolos ministeriais e municipais, os receituários de enfermagem são aceitos apenas nas farmácias vinculadas aos serviços públicos em ato administrativo específico, não sendo atendidos pelos serviços privados^(41)^. Para resolver esse impasse, o Distrito Federal aprovou o Projeto de Lei nº 574/2023, que obriga farmácias privadas a aceitarem prescrições de enfermeiros em programas de saúde pública e em rotina autorizada por instituições de saúde. No âmbito nacional, o Projeto de Lei nº 3.949/23, em tramitação, visa estender essa obrigatoriedade a todo o país e regulamentar pedidos de exames feitos por enfermeiros^(42)^. Essas são batalhas e conquistas que não fortalecem apenas a enfermagem, mas também o SUS, beneficiando diretamente a saúde pública e a população.

### Limitações do estudo

Na realização da pesquisa, enfrentaram-se dificuldades no recrutamento de participantes, o que exigiu a expansão dos locais de coleta e a colaboração de coordenações e outros envolvidos nos serviços para a divulgação. Outra limitação da pesquisa foi a não realização de coleta de dados com os usuários do sistema de saúde, o que impediu a compreensão da percepção desses sobre a ampliação da clínica do enfermeiro na APS e o impacto dos protocolos de enfermagem no seu cuidado. Portanto, recomenda-se que futuros estudos envolvam esse grupo populacional para obter uma visão mais ampla sobre o tema.

### Contribuições para a área da enfermagem

Acredita-se que este estudo possa contribuir para a enfermagem com o fortalecimento dos modelos de gestão do cuidado e promoção de práticas baseadas em evidências. Os resultados evidenciam a complexa interação entre protocolos de enfermagem e as macros e micropolíticas, ressaltando a necessidade de atualização contínua dos protocolos alinhada aos demais programas de saúde e políticas públicas.

## CONSIDERAÇÕES FINAIS

A relação entre as macro e micropolíticas para a gestão do cuidado na APS ocorre de modo complementar, por meio dos protocolos de enfermagem, em um processo dinâmico e interdependente em meio às interações entre profissionais, e os múltiplos aspectos da atuação em saúde. Reconhecer a relação entre as macros e micropolíticas da gestão do cuidado é fundamental para a enfermagem ampliar sua autonomia, representatividade e reconhecimento como categoria.

A gestão do cuidado na APS perpassa todas as dimensões, desde a individual até a societária, com a presença dos protocolos de enfermagem. O PACK utiliza os protocolos de enfermagem para seu aprimoramento, e ambos interagem mutuamente em um contexto onde tudo está interligado.

A ampliação da clínica do enfermeiro repercute diretamente na gestão do cuidado, e é conquistada via uso de protocolos de enfermagem na APS, entendendo que o uso destes não se faz de modo isolado, mas se faz na consulta do enfermeiro, por meio do processo de enfermagem, bem como subsidia a discussão clínica com outros profissionais.

## Data Availability

Os dados de pesquisa só estão disponíveis mediante solicitação.
